# Liver X Receptor Agonists Inhibit the Phospholipid Regulatory Gene CTP: Phosphoethanolamine Cytidylyltransferase-Pcyt2

**DOI:** 10.1155/2008/801849

**Published:** 2008-04-29

**Authors:** Lin Zhu, Marica Bakovic

**Affiliations:** Department of Human Health and Nutritional Sciences, University of Guelph, Ontario, Canada N1G 2W1

## Abstract

Metabolic pulse-chase experiments demonstrated that 25-hydroxycholesterol (25-OH), the endogenous activator of the liver X receptor (LXR), significantly reduced the biosynthesis of phosphatidylethanolamine via CDP-ethanolamine (Kennedy) pathway at the step catalyzed by CTP: phosphoethanolamine cytidylyltransferase (Pcyt2). In the mouse embryonic fibroblasts C3H10T1/2, the LXR synthetic agonist TO901317 lowered Pcyt2 promoter-luciferase activity in a concentration-dependent manner. Furthermore, 25-OH and TO901317 reduced mouse Pcyt2 mRNA and protein levels by 35–60%. The inhibitory effects of oxysterols and TO901317 on the Pcyt2 promoter function, mRNA and protein expression were conserved in the human breast cancer cells MCF-7. These studies identify the Pcyt2 gene as a novel target whereby LXR agonists may indirectly modulate inflammatory responses and atherosclerosis.

## 1. Introduction

Phosphatidylethanolamine (PE) is an essential membrane phospholipid with roles in multiple cellular processes including cell signaling, membrane fusion, cell division, autophagy, and apoptosis [1, 2]. Although PE could be synthesized by phosphatidylserine decarboxylation in mitochondria, the majority of PE is made de novo in the endoplasmic reticulum from ethanolamine and diacylglycerol via CDP-ethanolamine (Kennedy) pathway [1]. Molecular and metabolic aspects of the de novo Kennedy pathway and the role of the major regulatory enzyme Pcyt2 have been reviewed recently [2].

Liver X receptors (LXRs) are oxysterol-activated nuclear receptors, which control cholesterol homeostasis by modifying expression of genes involved in cholesterol absorption and efflux from peripheral tissues [3]. LXRs also regulate genes essential in lipogenesis, glucose metabolism, and inflammation [4]. Regulatory effects of LXRs on phospholipid genes are relatively unknown. Our initial characterization suggest that early growth response protein 1 and nuclear factor *κ*B (NF*κ*B) could be important for the regulation of the human Pcyt2 gene [2, 5]. Here, we report that oxysterols, 25-hydroxycholesterol (25-OH) and 22R-hydroxycholesterol (22R-OH), and the LXR synthetic agonist TO901713 downregulate the CDP-ethanolamine pathway and inhibit Pcyt2 gene expression by an indirect mechanism conserved in mouse and human cells.

## 2. Experimental procedures

**Cell maintenance and treatment**

Mouse embryonic fibroblasts C3H10T1/2 and human breast cancer cells MCF-7 grown under standard conditions [6, 7] were cultured 40–48 hours in a serum-free media supplemented with 25-OH (10 ng/*μ*L), 22R-OH (10 ng/*μ*L), or TO901713 (0–20 *μ*M) and the cells grown in serum-free media without agonists were used as controls.

**^14^C-ethanolamine radiolabeling and PE mass**

C3H10T1/2 cells (10^6^ cells/60mm-dish) were treated with 25-OH for 40 hours, pulse-labeled for 1 hour with ^14^C-ethanolamine (0.5 *μ*Ci/dish), chased with 250 *μ*M of “cold” ethanolamine and collected at different time points (0, 0.5, 1, 2, and 4 hours). The radio-labeled compounds were extracted by the Bligh-Dyer method and analyzed by TLC [6]. Total PE mass was measured using the fluorescent probe 1,6-diphenylhexatriene as we described previously [6].

**Pcyt2 promoter-luciferase reporter assays**

Transient transfections were performed as initially described [7]. The transfected cells were incubated for 5 hours in transfection media and then cultured in the presence or absence of LXR agonists for additional 48 hours or 15 hours. Luciferase reporter assays were performed using the dual luciferase system (Promega, Medison, WI, USA).

**Pcyt2 mRNA expression**

Total RNA was isolated with Trizol reagent (Invitrogen, Burlington On, Canada). PCR reactions were performed with 300 ng of single-stranded DNA using Pcyt2 specific primers, the forward primer F6 (5′ggagatgtcctctgagtaccg3′) and the reverse primer R7 (5′ggcaccagccacatagatgac3′). The primers produce two fragments of different size, 223 bp for Pcyt2*α* and 170 bp for Pcyt2*β* [8].

**Immunoblotting**

Cell homogenates were analyzed by western blotting using anti-Pcyt2*α* and anti-Pcyt2_(*α* + *β*)_ antibodies generated in our laboratory [6].

**Statistical analysis**

All measurements are expressed as means ±S.D. from at least three independent experiments. Data were analyzed by ANOVA (GraphPad Prism 3.0) and densitometry (Scion Image, Frederik, Maryland, USA).

## 3. Results

**CDP-ethanolamine (Kennedy) pathway is downregulated by 25-OH.**

**Figure 1:**
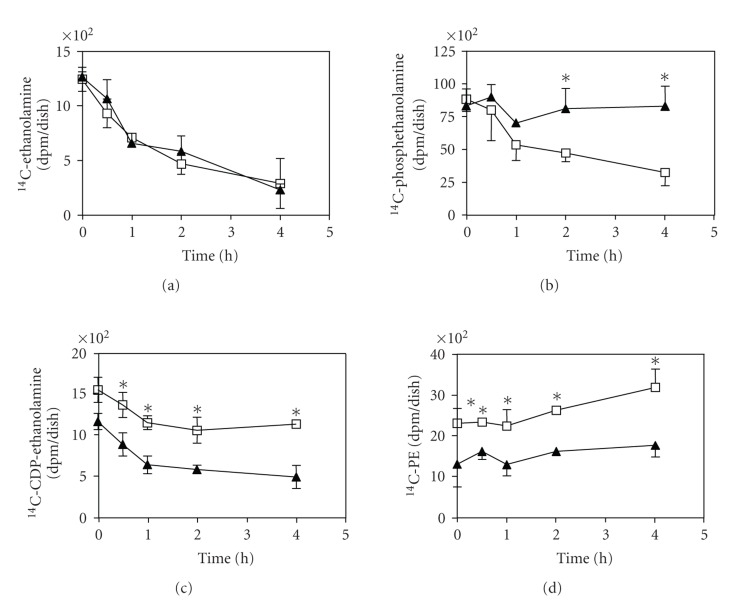
Inhibition of the CDP-ethanolamine pathway by 25-hydroxycholesterol. Mouse cells C3H10T1/2 were treated with 10 ng/*μ*L of 25-OH (filled triangles), “pulsed” with ^14^C-Etn for 1 hour and “chased” with an excess of unlabelled ethanolamine as indicated. ^14^C-ethanolamine (A), ^14^C-phosphoethanolamine (B), and ^14^C-CDP-ethanolamine (C) products were determined from the water phase and the radio-labeled PE (E) was quantified from the organic phase. Untreated cells grown in serum-free media (open squares) were used as controls. Data shown are from three independent experiments performed in duplicate; (*) indicates differences between treatments at *P* < .05.

The mouse embryonic cells C3H10T1/2 were treated with 10 ng/*μ*L of 25-OH oxysterol for 40 hours, radiolabelled with ^14^C-ethanolamine and chased with an excess of unlabeled ethanolamine for 0, 0.5, 1, 2, and 4 hours. As shown in Figure 1(a), the rate of ^14^C-ethanolamine disappearance was similar under both conditions, demonstrating that the ethanolamine kinase step (phosphoethanolamine formation) was not affected by the treatment. The formed ^14^C-phosphoethanolamine decreased from 8847 dpm at time 0 hour to 3246 dpm after 4-hour chase in untreated cells while in 25-OH treated cells ^14^C-phosphoethanolamine disappeared very slowly (Figure 1(b)), suggesting that this step catalyzed by Pcyt2 was inhibited by the oxysterol treatment. Consequently, the Pcyt2 product ^14^C-CDP-ethanoalmine remained constantly low in 25-OH treated cells, and the difference between ^14^C-CDP-ethanoalmine in 25-OH treated and control cells reached 64% at the end of the chase (Figure 1(c)). The rates of disappearance of ^14^C-CDP-ethanoalmine were on the other hand similar, demonstrating that the last step in the Kennedy pathway (phosphotransferase step) was not affected by the oxysterol treatments (Figure 1(c)). The slower formation of CDP-ethanolamine in 25-OH treated cells was accompanied with significantly reduced rate of ^14^C-PE synthesis (Figure 1(d)). Under the same conditions the total PE decreased by ∼32% in the cells treated with 25-OH (not shown). Taken together, these data demonstrate for the first time that the PE de novo synthesis via the Kennedy pathway became downregulated by 25-OH at the step of CDP-ethanolamine formation, which is catalyzed by Pcyt2.

**LXR agonists downregulate mouse Pcyt2 promoter and gene expression**

**Figure 2:**
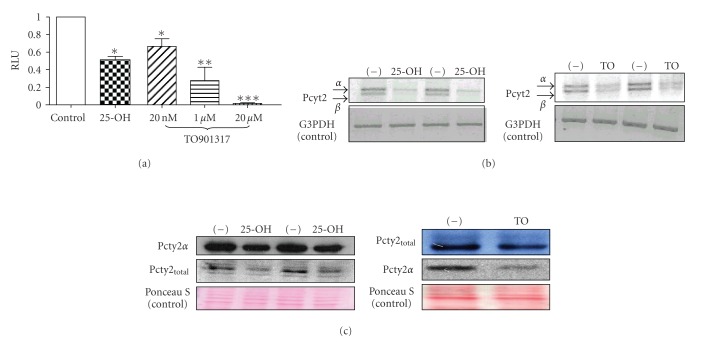
Downregulation of the mouse Pcyt2 gene by 25-hydroxycholesterol and the LXR-specific agonist TO901317. (a) C3H10T1/2 cells were cultured in the presence of 25-OH (10 ng/*μ*L) or TO901317 (20 nM–20 *μ*M) for 48 hours. Shown are promoter-luciferase reporter activities from four independent experiments performed in duplicates. The numerical values represent means ±S.D., with significant differences indicated as (*) at *P* < .05, (**) at *P* < .01 and (***) at *P* < .001. (b) Pcyt2 mRNAs (*α* and *β*) determined in 25-OH (*left panel*) and 1 *μ*M TO901317 (*right panel*) treated cells. (c): Western blot showing that 25-OH (*left panel)* and 1 *μ*M TO901317 (*right panel*) treatments of C3H10T1/2 cells reduced total (*α* + *β*) and *α* Pcyt2 proteins.

To establish the mechanism for the 25-OH effect on Pcyt2, we performed luciferase-reporter assays using the previously characterized mouse Pcyt2 promoter (−559/+29 bp) [7]. As shown in Figure 2(a), 25-OH reduced promoter activity 40% (P < .05) at the concentration of 10 ng/*μ*L after 48 hours treatments. To test whether the inhibitory effect on Pcyt2 was through the activation of LXR, the C3H10T1/2 cells were also treated with the specific LXR agonist TO901317, and the inhibitory effect of TO901317 on the mouse Pcyt2 promoter was dose-dependent at 0.02–20 *μ*M (Figure 2(a)). The reduction of promoter function was accompanied by dramatically reduced expression of both Pcyt2*α* and -*β* transcripts (Figure 2(b)). Using specific antibodies made in our laboratory for total Pcyt2 (*α* + *β*) and for Pcyt2*α* proteins, we further established that both total Pcyt2 (*α* + *β*) and its *α* form were significantly reduced by the 48 hours treatments of 25-OH and 1 *μ*M TO901317 (Figure 2(c)). These data demonstrate that 25-OH and LXR specific ligand TO901317 had similar inhibitory effects on the Pcyt2 gene expression. When we measured the effects of 25-OH and TO901317 on Pcyt2 gene expression after 15-hour treatments, the lowering effects on promoter activities and Pcyt2 protein amounts were as significant as 48 hours treatments (data not shown).

**LXR agonists downregulate human Pcyt2 promoter and gene expression**

**Figure 3:**
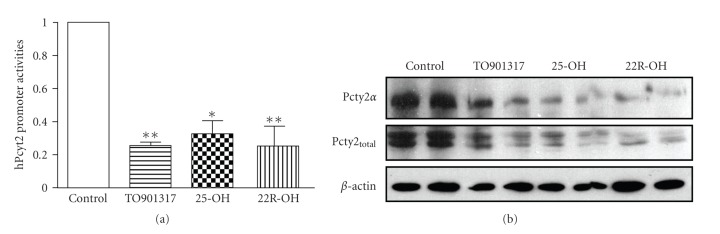
LXR agonists attenuate the expression of the human Pcyt2 gene. (a) The human breast cancer cells MCF-7 were transfected with the human Pcyt2 promoter-luciferase reporter construct and treated with TO901317 (1 *μ*M), 25-OH (10 ng/*μ*L) or 22(R)-OH (10 ng/*μ*L) for 48 hours. Luciferase activities for untreated and treated cells were performed. Shown are means ±S.D. of at least in four independent experiments in duplicate, and significant differences are indicated as (*) at *P* < .05, (**) at *P* < .01, and (***) at *P* < .001. (b) Attenuation of total and *α* Pcyt2 proteins after various treatments as in (a).

The effect of LXR agonists on the Pcyt2 gene was also tested in human cells. The human breast cancer cells MCF-7 were transiently transfected with the human Pcyt2 promoter luciferase reporter (−590/+56 bp) [7] and treated with TO901317 (1 *μ*M), 25-OH (10 ng/*μ*L) or 22R-OH (10 ng/*μ*L). As shown in Figure 3(a), TO901317 reduced the human Pcyt2 promoter activity by 76% (*P* < .05), and oxysterols 25-OH and 22R-OH decreased the luciferase activity, respectively, by 52% (*P* < .05) and 63% (*P* < .05). In agreement with the effect on the promoter activity, TO901317, 25-OH and 22R-OH were also able to considerably reduce the total Pcyt2(*α* + *β*) and Pcyt2*α* protein levels in treated cells relative to untreated MCF-7 cells (Figures 3(b)).

## 4. Discussion

In this report, we demonstrate for the first time that natural oxysterols and the LXR synthetic agonist T0901317 are inhibitors of the PE de novo synthesis at the step of CDP-ethanolamine formation by downregulating the Pcyt2 gene at the transcriptional level. Recently, it has been demonstrated that oxysterols could also inhibit phosphatidylcholine (PC) *de novo* synthesis by blocking the phosphorylation of the related enzyme, CTP: phosphocholine cytidylyltransferase-Pcyt1, in the choline branch of the Kennedy pathway [9]. That oxysterols and LXR agonists inhibit PE and PC indicates that they are important regulators of the membrane biogenesis at the level of the two major phospholipids.

LXRs are best-known for their ability to modulate cholesterol efflux by ABCA1 [3] and it is established that reduced HDL phospholipids (PC and PE) could enhance the ABCA1-mediated efflux and reduce the SR-BI-mediated efflux [10]. Therefore, a reduced rate of PE synthesis and for the same matter reduced PC synthesis by the LXR could potentially lead to lower phospholipid (PE and PC) availability for serum lipoproteins, thereby favoring the ABCA1-mediated cholesterol efflux over the SR-B1-mediated cholesterol efflux. In addition, reduced cellular PC and PE, due to inhibition of Pcyt2 and Pcyt1, could also limit the extent of cholesterol efflux to ApoA1 or HDL since the transport of phospholipids and cholesterol are linked.

LXRs and their ligands are well-established negative regulators of the proinflammatory genes including COX1/2 and prostaglandin E synthase-1 (PGES-1) [4, 11]. PE and PE-plasmalogens are the major sources of arachidonic acid, the principal substrate of the prostanoid inflammatory mediators [12, 13]. COX1 and COX2 convert arachidonic acid released from PE and PC into prostaglandin H2, which is a sole substrate for a series of other prostaglandins. Based on our findings, the anti-inflammatory potency of the LXR agonists appears to inhibit the phospholipid (PE and PC) synthesis to reduce the arachidonic acid reservoir pool, in addition to their known effect of suppressing the arachidonic acid utilization by COX1/2 and downstream genes.

Anti-inflammatory properties of LXR are mostly mediated indirectly by transactivation of other transcription factors such as NF*κ*B and Ap1(c-Fos/c-Jun) [3, 14]. Our thorough analysis of the mouse and human Pcyt2 promoter sequence did not reveal any conserved LXR response elements [7], suggesting that the observed inhibitory action of the LXR agonists on the Pcyt2 transcription is also indirect. We have already established that human Pcyt2 could be regulated by NF*κ*B [5], but the mouse form is not an NF*κ*B target [7] and the LXR agonists inhibit both mouse and human promoters. The mouse and human Pcyt2 promoters on the other hand share several putative Ap1 [7] and glucocorticoid receptor (GR) response elements (data not shown), which could potentially be involved in the LXR inhibitory action. It is established that the LXR agonist T0901317 markedly suppresses the GR gene and its downstream targets involved in hepatic glucose metabolism and therefore ameliorates diabetic syndrome in db/db mice [15].

In conclusion, we established that oxysterols and the LXR specific agonist T0901317 diminish Pcyt2 promoter and gene expression using an indirect mechanism that is conserved in mouse and human cells. Because LXR agonists inhibiting PE synthesis may contribute to their effects in cholesterol homeostasis and inflammation, to suppress the de novo PE synthesis by inhibiting Pcyt2 could be an alternative choice for developing such therapeutics.
